# Novel Vaccines to Human Rabies

**DOI:** 10.1371/journal.pntd.0000515

**Published:** 2009-09-29

**Authors:** Hildegund C. J. Ertl

**Affiliations:** The Wistar Institute, Philadelphia, Pennsylvania, United States of America; Centers for Disease Control and Prevention, United States of America

## Abstract

Rabies, the most fatal of all infectious diseases, remains a major public health problem in developing countries, claiming the lives of an estimated 55,000 people each year. Most fatal rabies cases, with more than half of them in children, result from dog bites and occur among low-income families in Southeast Asia and Africa. Safe and efficacious vaccines are available to prevent rabies. However, they have to be given repeatedly, three times for pre-exposure vaccination and four to five times for post-exposure prophylaxis (PEP). In cases of severe exposure, a regimen of vaccine combined with a rabies immunoglobulin (RIG) preparation is required. The high incidence of fatal rabies is linked to a lack of knowledge on the appropriate treatment of bite wounds, lack of access to costly PEP, and failure to follow up with repeat immunizations. New, more immunogenic but less costly rabies virus vaccines are needed to reduce the toll of rabies on human lives. A preventative vaccine used for the immunization of children, especially those in high incidence countries, would be expected to lower fatality rates. Such a vaccine would have to be inexpensive, safe, and provide sustained protection, preferably after a single dose. Novel regimens are also needed for PEP to reduce the need for the already scarce and costly RIG and to reduce the number of vaccine doses to one or two. In this review, the pipeline of new rabies vaccines that are in pre-clinical testing is provided and an opinion on those that might be best suited as potential replacements for the currently used vaccines is offered.

## Introduction

Rabies virus has a relatively simple RNA genome that encodes five structural proteins. Of these, the rabies virus glycoprotein is the only target for neutralizing antibodies (NAs), which provide full protection against virus challenge [1]–[3]. Due to efforts spearheaded by the World Health Organization (WHO), standardized assays to measure NA titers to rabies virus are available [4]. Titers equal to or more than 0.5 international units (IU), determined by an infectious foci reduction assay against a WHO reference serum, are considered protective in mammalian species tested to date.

Rabies virus has the highest fatality rate of all known human viral pathogens. With less than a handful of exceptions, humans that develop a symptomatic rabies virus infection inevitably die. Most of the few survivors had extensive brain damage following the infection [5]. One survivor receiving a novel treatment based on drug-induced coma survived without long-term neurological damage [6]. Nevertheless, in subsequent studies this treatment failed to affect the outcome of the disease in other rabies patients [7].

Humans are exposed to rabies, in general, through a bite by a rabies virus–infected animal or through mucosal contact with virus-contaminated fluids. In the United States, due to mandatory pet vaccinations and public awareness of the potential to transmit rabies virus through wild carnivores and bats, rabies virus infections in humans are rare. In fact, the few human rabies infections that are reported each year are caused by rabid bats [8],[9]. These transmissions, which generally result from minor skin abrasions, are often overlooked. In developing countries, human rabies, most commonly transmitted through the bite of rabid dogs, is far more prevalent, causing 25,000–30,000 deaths each year in India alone [10]. Half of the infections occur in children. In other Asian countries, such as China, the incidence of rabies is increasing [11]. Incidence rates are largely unknown for Central Africa due to a lack of incidence reporting [12]. In developing countries, dogs are commonly ownerless or community owned and not vaccinated. Programs to vaccinate, sterilize, or euthanize stray dogs have been attempted in these countries but have generally failed [13]. Euthanasia of dogs is largely ineffective since a decrease in the population results in increased breeding of the remaining animals. Vaccination of the animals is only effective if ∼70% of the dogs are vaccinated. Considering the large numbers of stray dogs in countries such as India and Thailand, continued vaccination of stray dogs poses major logistical problems that are nearly insurmountable [14]. For the same reason, sterilization of high enough numbers of stray dogs to reduce their population has been impossible. Thus, rabies virus continues to spill over into the human population [10].

Efficacious rabies vaccines for humans are commercially available. In developed countries, the vaccines are based on fixed strains of rabies virus, such as the Pitman Moore (PM) strain, the Kissling strain of Challenge Virus Standard (CVS), or the Flury low egg passage (LEP) fixed rabies virus strain [15]. These vaccine preparations contain inactivated virus and some of them contain adjuvant. They have to be given three times in a prophylactic treatment to achieve protective immunity, which then, in general, lasts for 3–4 years. Upon exposure to rabies virus, the vaccines have to be given four to five times [15],[16], and in cases of severe exposure they have to be combined with rabies virus–specific immunoglobulin (RIG). RIG, which should be of human origin (HRIG), is in limited supply and plans to replace it with monoclonal antibody preparations are ongoing [17]. Equine rabies immunoglobulin (ERIG) preparations or purified F(ab)2 preparations of ERIG, which are digested antibodies from which the immunogenic constant region is removed but which retains binding to antigen, are also available. ERIG can cause serious side effects including anaphylactic reactions and serum sickness. The F(ab)2 preparation is better tolerated [18] but has been linked anecdotally to vaccine failures, which may relate to the relative short half-life of F(ab)2 fragments. The current post-exposure prophylaxis (PEP), although highly efficacious and generally tolerated, is very costly (approximately US$800–US$1,500 for treatment including RIG) and requires the patient to seek medical treatment four to five times.

Some of the less developed countries still use the Semple vaccine derived from rabies virus propagated in the brains of infected sheep or goats [19]. The Semple vaccine, in which the virus is inactivated by phenol treatment, requires 14–21 daily injections. The Semple vaccine is cheaper than vaccines produced from cell or tissue culture but it has a potential for serious side effects in the form of vaccine-induced auto-immune encephalomyelitis, which afflicts ∼1∶200 vaccinees [20].

In part, the high incidence of rabies in spite of effective vaccines is linked to ignorance of appropriate treatment [21]. Adults bitten by a dog or parents of children bitten by a dog fail to appreciate the potential for infection with a fatal virus. In some of the Asian countries, patients are still seeking alternative treatments such as herbal remedies to ward off a rabies virus infection. Otherwise, vaccine failures are a consequence of inappropriate PEP caused either by the health care provider's lack of knowledge or by financial restraints on the patient or his/her parents, the latter being demonstrated by the fact that most rabies-related deaths in India occur among economically disadvantaged families. In India, wound cleaning is only performed in ∼40% of patients that present with a dog bite [22]. As already mentioned, RIG is costly and in very short supply, and is thus commonly omitted. Most of the RIG has to be given locally into the bite site and again, this is not always done. The vaccine, depending on the regimen, has to be given four to five times over 28 days. Often patients lack the resources to seek medical attention repeatedly and thus only receive the initial dose. Overall, in India only 47% of patients that require vaccination receive it [22]. Again in India, a dog bites a human every 2 seconds and every 30 minutes a human dies of rabies [22]. The actual number may be by far higher as rabies is not a reportable disease in India. In India, the annual cost due to person-days lost because of animal bites is US$38 million, and the cost of PEP is about US$25 million. A human that receives vaccination after exposure loses, on average, 2.2 days of income [22],[23].

Considering the severity of rabies and its continued high incidence in developing countries, development of novel vaccines for rabies is warranted. A preventative vaccine used for immunization of children, especially those is high incidence countries, would be expected to lower fatality rates. Such a vaccine would have to be inexpensive and provide sustained protection, preferentially after a single dose application. For PEP, novel regimens are needed that allow for a reduction of the scarce and costly RIG and a lowering of the number of vaccine doses.

## Methods

To compile this review, literature has been selected from PubMed searches. Considering the multitude of manuscripts that describe the new vaccine platforms, only a limited number of them could be cited. Emphasis is given to reports that used standardized assays to assess vaccine immunogenicity in comparison to traditional vaccines, and that tested efficacy of preventative vaccination or PEP in challenge models. In the end, the type(s) of vaccine that is (are) the most promising of the candidates to replace current regimens, is (are) the one(s) that outperforms all current vaccines.

## Vaccines to Rabies Virus

Traditionally, rabies vaccines have been generated by serial passage of rabies virus in brain tissue or cell culture until attenuated (live virus) or by inactivation of the virus (killed virus). With advances in molecular virology, it is now possible to create attenuated virus vaccines through genetic engineering, or to purify viral antigens from highly efficient protein expression systems, or to express individual viral antigens by inserting their genes into different vaccine carriers. This has allowed the exploration and analysis of immune responses to individual viral antigens, which in turn has uncovered those most suited to induce protective immunity. In the case of rabies virus, the prime immune correlate of protection against the virus is the induction of NAs, and the only viral antigen capable of achieving this is the rabies virus glycoprotein. Accordingly, subunit vaccines that express this antigen provide full protection to viral challenge [24],[25]. Different novel vaccines for rabies have been developed recently and their advantages and disadvantages for use in humans are discussed below.

### Rabies Virus Vaccines Based on Reconstituted and Traditionally Attenuated Viruses

Traditionally attenuated rabies viruses have been used successfully for oral immunization of animals in the wild that consume baits containing the vaccine. A number of attenuated viral strains have been used in baits for oral vaccination. All were derived from a common ancestor, i.e., the Street Alabama Dufferin (SAD) [26], including the Evelyn Rokitniki Abelseth strain, which has been used, in particular, for vaccination of foxes [27]. This strain, however, was found to cause fatal rabies in cats [28]. Traditional rabies virus attenuation, in general, is linked to the amino acid sequence of the viral glycoprotein (G). For example, a mutation in position 333 of the glycoprotein where arginine is replaced by glutamic acids generally attenuates the virus and provides the basis for the apathogenic SAD strains [29]. Other mutations between amino acid residues 164–303 can also reduce pathogenicity in some strains [30]. Attenuation by a single amino acid exchange, however, may not guarantee a stable nonpathogenic phenotype of a rapidly mutating RNA virus. Indeed, it was found that rabies virus attenuation through an amino acid substitution for Arg333 can revert back to pathogenicity upon serial passage of the virus in mouse brain through the introduction of mutations at other positions of the glycoprotein gene [31].

Advances in reverse genetics to produce reconstituted rabies virus have allowed for targeted alterations of the genome of rabies virus through expression of mutated genes. Using reverse genetic techniques, a number of attenuated rabies viruses have been generated and tested. One group developed a rabies virus based on the Flury high egg passage (HEP) strain in which the gene encoding the non-catalytic phosphoprotein (P) cofactor of the viral polymerase was deleted. The resulting P-deleted virus was able to grow in cell lines that constitutively provide the missing P antigen for virus assembly and propagation. A P-deleted virus that cannot replicate in cells is completely apathogenic. In mice, reconstituted rabies virus lacking the P gene induces a strong NA response that protects against challenge [32]. Similarly, deletion of the matrix protein (M)-encoding gene from the RC-HL strain of reconstituted rabies virus resulted in an apathogenic virus that retained its full immunogenicity in relation to the glycoprotein [33]. The immunogenicity of such deletion mutants was increased further by insertion of an additional glycoprotein gene [34]. In mice, antibody titers came up faster and the response was dominated by antibodies of the IgG2a isotype, which is indicative of a T helper cell type 1 (TH1) response, unlike the TH2-linked IgG1 response induced by the inactivated wild-type viral vaccine. Interestingly, protection of mice was achieved with a single moderate dose of the attenuated viruses that carried two glycoprotein genes. Again, it should be stressed that the P-deleted and M-deleted viruses were completely apathogenic even in immunodeficient animals. A recent publication describes a triple glycoprotein variant rabies virus that yielded promising results in mice [35]. It remains to be shown that reconstituted mutant viruses expressing two or three glycoprotein molecules are stable and that yields from large-scale production are similar to those achieved with traditional vaccine strains. Biodistribution studies have to be conducted to ensure that the mutants do not reach the central nervous system and cause neurotoxicity in larger mammals. Provided that the double or triple glycoprotein expressing rabies virus mutants pass these tests and show safety and immunogenicity in clinical trials, they would be excellent candidates for both pre- and post-exposure immunization to rabies. Moreover, they would offer a number of cost-saving advantages. The vaccine would not need to be inactivated, which should reduce cost. In pre-exposure situations, the vaccine would be expected to induce protective titers of NAs after a single dose rather than after the three doses that are required for currently available rabies vaccine. And finally, the more rapid the onset of a response, the greater the possibility that the vaccine may allow for a reduction in the use of RIG.

### Novel Adjuvants

Commercially available rabies vaccines have to be given repeatedly for prevention as well as for PEP, which adds to the cost of rabies vaccination, both in terms of biologicals and expenses associated with seeking medical treatment. An adjuvant that would increase the immunogenicity of the vaccine and allow for a reduction of numbers of doses would thus be very useful to lessen the cost and reduce vaccine failures due to incomplete vaccinations. Commercially available inactivated rabies virus vaccines either do not contain adjuvant or are adjuvanted with alum, which promotes a TH2 response favoring induction of antibodies of the IgG1 isotype in mice [36]. In the last decade, our understanding of basic immunological pathways that promote immune responses has increased tremendously and we have learned that activation of innate immune responses is a crucial first step in the induction of adoptive immune responses. Signals involved in activation of cells of the innate immune cells have been identified, such as membrane-bound Toll-like receptors (TLRs) and cytoplasmic NOD-like receptors [37]. This in turn has led to the development of new types of adjuvants to replace alum, which is well tolerated but not overly potent. Examples for such novel adjuvants are CpG oligodeoxynucleotides (ODNs), which activate the innate immune system upon binding to TLR-9. One group tested the inactivated rabies vaccine adjuvanted with alum in comparison to one adjuvanted with a CPG-ODN [38]. Three doses of the latter induced rabies virus NA titers in mice that were comparable to those induced with five doses of the alum-adjuvanted vaccines. Although these results are promising, a word of caution is needed. Mice and humans differ in their expression pattern of TLR-9; results obtained in mice may thus not necessarily translate to humans. If, on the other hand, human trials show a dose-sparing effect of the already commercially available vaccine preparations upon replacement of alum with CpG-ODNs, such preparations clearly would be prime candidates to replace currently used vaccines. In recent years, a number of new adjuvants have been licensed or are in pre-clinical development [39]. Their mode of action is being elucidated [40], thus allowing for a more targeted formulation of vaccines. Formulation of rabies vaccines with other second generation adjuvants should be explored.

### Protein Subunit Vaccines

A number of groups have tested vaccines administered orally that are based on rabies virus glycoprotein purified from virus [41], or expressed in insect cells infected with recombinant baculovirus vectors [42], in yeast cultures that express the viral glycoprotein [43], in glycoprotein cDNA-transfected cells [44], or in transgenic plants [45]. These experiments met with variable success. This is not surprising considering the structural complexity of the rabies virus glycoprotein, which carries two branches of N-linked oligosaccharides [46]. Most of the rabies virus NAs bind to conformation-dependent epitopes on the native glycoprotein that appear to be expressed preferentially by multimeric forms of the protein, rather than by monomeric forms of cleaved glycoprotein that is secreted by infected cells or that is commonly produced by expression systems. Although yeast has been used successfully to develop proteins such as hepatitis B virus surface antigen vaccine, expression of the rabies glycoprotein in yeast resulted in an incorrectly folded protein that was only poorly immunogenic [43].

Upon infection with a recombinant baculovirus, insect cells produced a rabies glycoprotein that was antigenically conserved compared to the native protein, although the recombinant glycoprotein was slightly smaller in size, suggesting it might have different post-translational modifications. Immunization of mice with rabies glycoprotein–producing insect cells caused induction of protective NA titers [42]. In raccoons, repeated oral immunization with rabies virus glycoprotein–containing cell lysate induced rabies virus NA titers that protected most but not all of the animals from lethal challenge [47]. For human vaccination, the use of cells or cell lysate would not be appropriate. Purification of glycoproteins is formidable and cumbersome, often ineffective, and costly. Although purified protein vaccines are in general well tolerated, it is unlikely that a baculovirus-derived rabies glycoprotein vaccine would be less costly than current vaccines.

Other investigators have focused on expressing the rabies virus glycoproteins in plants. The rabies virus glycoprotein expressed within tobacco leaves was shown to be authentically glycosylated. It was also immunogenic and induced protection in intramuscularly immunized mice [48]. Similarly, a single oral immunization of mice with transformed maize kernels containing rabies glycoprotein induced protection against a lethal challenge [49]. Production of medicinal proteins in plants has the clear advantage that large amounts of protein can be generated at a low cost. For the generation of a vaccine to be given by injection, the cost of producing the protein in plants is offset by a need for purification of the protein at relatively high cost. Intuitively, far more attractive is the concept to generate edible vaccine antigens expressed by vegetable or fruits that are part of the human diet. Techniques have been developed to generate transgenic bananas, potatoes, tomatoes, spinach, and the like that express foreign proteins, and these techniques are being refined to ensure more authentic post-translational modifications. Oral immunization has been successful with whole or intact viruses that typically resist degradation in the digestive tract. One prime example is the live poliovirus vaccine that was given to children on lumps of sugar. Other less hardy viruses, such as the adenovirus (Ad) vaccines that were given to the US military, had to be encapsidated to protect them against acid degradation and to retain immunogenicity [50]. Oral rabies vaccines that are given in baits to raccoons, foxes, and other free-ranging animals do not induce immune responses in the intestine but rather infect cells of the oral cavity, which is rich in lymphatic tissues, e.g., tonsils [51]. Although this was not investigated in detail, glycoprotein-containing insect cells or plant cells presumably also require presentation of the antigens by cells of the oral cavity. Would it be expected that successful oral immunization of animals readily translates to humans? Considering that humans vary widely in their eating habits, similar to animals—some chew carefully, others gulp down big chunks of food—the simple feeding of rabies virus glycoprotein-containing plants to achieve protective immunity against rabies is unlikely to yield uniform responses. One also needs to consider how the immune system may react if some of the antigen successfully passes the stomach, as could happen in humans with low stomach acid. The intestinal immune system, which is targeted by antigens that pass through the stomach, is substantially different from the systemic immune system, which responds to antigens that are directly injected into muscle or skin. The intestinal immune system is constantly bombarded by harmless antigen present in food in addition to harmful antigens of pathogens. Thus, the intestinal immune system has to balance between tolerating beneficial antigens while still combating pathogens. Proteins present in the intestine are viewed as beneficial and thus induce systemic unresponsiveness, a phenomenon termed oral tolerance [52]. If and to what degree an edible rabies virus glycoprotein vaccine may induce oral tolerance clearly needs to be investigated before such vaccines can even enter clinical trials for oral immunization of humans.

As a rather clever variation of edible vaccines, another group developed plant hybrid viruses based on alfalfa mosaic virus or tobacco mosaic virus [53]. The coat proteins of these viruses were genetically modified to express a peptide composed of antibody binding sequences of the rabies virus glycoprotein and the nucleoprotein. Although these vaccines are not classical protein vaccines, they are included in this part of the review since they represent virus particles that carry and present the rabies virus antigen on their surface, unlike recombinant viral vector vaccines that carry the genetic information of rabies virus. The viruses were propagated in tobacco or spinach plants, respectively, and then purified and given three times to mice. The mice fed the plant-derived protein were protected against rabies virus challenge. The vaccine produced in spinach was also tested in humans. Some of the human volunteers that already had NAs to rabies virus showed a slight increase in NA titers upon repeated ingestion of virus-containing spinach leaves. Seronegative humans did not develop detectable titers of rabies virus NAs after being fed several doses of the spinach vaccine, although three out of nine appeared to have been primed as they developed higher than expected NA titers after inoculation with one dose of a commercially available rabies virus vaccine [53]. Overall, this approach, although intellectually very appealing, needs to be refined substantially before it becomes a viable option for an alternative rabies vaccine.

### DNA Vaccines

A large number of publications that address the development of novel rabies vaccines focus on the use of plasmid vectors, also termed DNA vaccines [54]–[57]. DNA vaccines are appealing for many reasons: they are extraordinarily stable, they only induce immune responses to the wanted antigen, and they carry their own adjuvant in the form of unmethylated CpG sequences present in the bacterial backbone of the vector. They are also easy to generate and their scale-up production is more uncomplicated and cost effective than that of purified protein vaccines or vaccines that require mammalian tissue cell culture. DNA vaccines are safe, they do not integrate into the host cell genome, they do not induce antibodies to DNA or autoimmune diseases, and they only elicit mild local reactions, as shown in numerous clinical trials. Overall, results have shown that immunization with plasmid vectors resulted in the slow and modest induction of rabies virus NAs. Responses to one dose of plasmid vector were well below those to one dose of a traditional inactivated rabies virus vaccine [57]. Responses could be improved by repeated immunization [57], by the use of adjuvants such as other vectors encoding cytokines [58], or monophosphorolipid A [59]. Responses were increased by intradermal rather than intramuscular immunization or by application of the vector with a gene gun, which propels DNA-loaded gold particles directly into the cells of the skin [59]. DNA vaccines were tested in larger species and reports showed protection of dogs and cats with DNA vaccines given at moderate doses [60],[61]. One group showed induction of NA responses and protection against challenge in nonhuman primates [62]. Others have explored the use of replicon-based self replicating DNA vaccines derived from an alphavirus, i.e. Sindbis virus [63]. Although rabies virus NA responses to the replicon DNA vaccine given twice exceeded those to a conventional DNA vaccine, they did not reach the levels obtained by two doses of a conventional whole virus vaccine.

One group reported on successful post-exposure vaccination of rabbits and mice with DNA vaccines given intranasally [64]. In this report, all of the animals that received the DNA vaccine were protected, while one out of eight rabbits that received a commercial human rabies vaccine died. Similar results were obtained for PEP of mice. These results have to be viewed with caution. The plasmid DNA was found in numerous areas of the brain after immunization and the authors concluded that the local immune response initiated within the brain by the DNA vaccine was linked to the observed efficacy of the vaccine. Adaptive immune responses are not initiated in the brain but rather in lymph nodes, which are not present in the central nervous system. The presence of DNA vectors in the central nervous system in the study is worrisome and difficult to understand. The intranasal immunization with the DNA vaccine was controlled for by an intramuscular injection of saline. This is not an appropriate control, as it does not take into account DNA vaccine-driven activation of the innate response that may have contributed to protection. Post-exposure vaccination of nonhuman primates, combined with RIG, resulted in partial protection [65].

In spite of these two reports, the slow onset of an immune response to the transgene product of a DNA vaccine observed by numerous investigators using different routes of administration precludes their use for post-exposure immunization, when immune responses have to be induced rapidly to ward off a symptomatic rabies virus infection of the central nervous system. Are they useful for preventative vaccination to reduce the incidence of rabies infections in highly endemic resource-poor regions? A number of DNA vaccines expressing antigens from other viral pathogens, which can be prevented by NAs, have undergone clinical testing. Examples are DNA vaccines expressing the hemagglutinin of influenza A virus or the surface antigen of hepatitis B virus [66]. Results were uniformly disappointing; the DNA vaccines applied in various ways, including injection by syringe or administration by gene gun, either failed to induce a detectable antibody response or the antibody response was very low. Lack of uptake of DNA vaccines by cells at the inoculation site has been identified as a barrier to efficient antigen production in vivo, which in turn is needed for an optimized immune response. DNA uptake can be improved technically by applying short electrical pulses, i.e., electroporation, to the DNA vaccine–injected muscles. DNA vaccination combined with electroporation has yielded very promising results in experimental animals, in which antibody titers induced by this method exceed those induced by simple injection of a DNA vaccine by nearly 1,000-fold [67]. Several clinical trials have been started to assess the effectiveness of electroporation of DNA vaccines in humans; results are not yet available. Hence, it remains to be established if DNA vaccination by electroporation is effective for prevention of rabies in humans.

### Recombinant Viral Vector Vaccines

Viral vectors, based on a variety of different parental viruses carrying the gene for the rabies virus glycoprotein under a suitable promoter, have been explored as vaccine carriers. Although viral vector vaccines offer many advantages, including faithful expression of foreign viral antigens in mammalian cells, they have one disadvantage. That is, existing NAs to the parental virus in the target species can inhibit the uptake of recombinant viral vectors and hence production of the vaccine antigen. This reduces, or in the extreme even abolishes, expression of the transgene product and thus impairs the immunogenicity and efficacy of the viral vector vaccine. Many viral vectors are based on pathogens that are common in the target species. For example, vectors based on the human adenovirus serotype 5 (AdHu5) have undergone extensive testing as a vaccine carrier in humans [68], while those based on canine adenovirus 2 are being explored for use in dogs [69]. Both species commonly have NAs to either virus [70],[71]. The use of vectors based on common pathogens needs to be explored in the presence of vector-specific NAs to gain a realistic assessment of their immunogenicity in their target species.

### Poxvirus Vectors

A vaccinia virus recombinant expressing the rabies virus glycoprotein (VRG) is being used commercially for oral immunization of wildlife. The recombinant is based on the highly reactogenic Copenhagen strain of vaccinia virus, which is not suited for vaccination of humans [72]. More attenuated poxviruses have been developed, such the modified vaccinia virus Ankara (MVA), which upon numerous passages in cells deleted ∼15% of the genome of the parental virus. MVA is replication incompetent in most cells and it is well tolerated, even by humans that have underlying conditions that would preclude them from vaccination with vaccinia virus. MVA vectors expressing the rabies virus glycoprotein were tested in mice [73]. Results clearly showed that the MVA vectors were less immunogenic than the VRG vaccine. In dogs and raccoons that already had NAs to rabies virus, the MVA vector induced an anamnestic response if given intramuscularly, but was ineffective upon oral administration. Overall, these results suggest that MVA vectors are not suited to replace currently available conventional or recombinant vaccines for use in animals or humans.

### Herpesvirus Vectors

A vector based on an attenuated porcine herpesvirus 1, also termed pseudorabies virus, expressing the rabies virus glycoprotein was tested in dogs [74]. Pseudorabies virus causes abortions in sows and a fatal disease in piglets. An attenuated virus, used as a vaccine in pigs, is efficacious and safe. Dogs and cats can become infected with pseudorabies virus and the infection in these species is commonly fatal. As pigs are the only reservoir for pseudorabies virus, prevalence rates of NAs to this virus that may interfere with a recombinant pseudorabies virus-based vaccine would be expected to be low in other species, such as dogs or humans. Oral immunization with the pseudorabies virus vaccine expressing the rabies virus glycoprotein resulted in protective antibody titers in dogs. Nevertheless, although the vaccine virus was applied at high doses, NA titers were lower than those obtained with a conventional vaccine [74].

### Ad Vectors

A multitude of Ads have been isolated from different species including humans, chimpanzees, other nonhuman primates, bovines, canines, birds, snakes, and frogs. Phylogenetic analysis showed that Ads can be grouped into three major trees that share common ancestors [75]. Human Ads are closely related to those isolated from chimpanzees. They are also closely related to Ads isolated from dogs, mice, pigs, and horses. Ads isolated from bovines fall into two trees; some are related to human Ads, others are more closely related to those isolated from goats, sheep, deer, and ducks. Ads isolated from fowl are grouped with those isolated from amphibians [75]. Ads are subdivided not only according to the species from which they originated, but also into families, such as families A–E for human Ads, and into serotypes, which are defined by the specificity of NAs.

Ad vectors derived from different serotypes and species have been tested extensively as vaccine carriers for a variety of different pathogens. Ads are ubiquitous pathogens and the prevalence of NAs to the more common serotypes is high in humans as well as animals. NAs inhibit or weaken infection of the host cells by the corresponding Ad or by a vaccine vector based on this Ad, resulting in reduced immune responses to the encoded antigen. Ad vectors based on serotypes that are common in a given target species should thus not be used as vaccine carriers in this species, as the desired immune response will be highly variable depending on the presence and magnitude of Ad-specific NAs in the vaccine recipient.

### Ad Vectors Based on the Human Serotype 5 (AdHu5)

Most vaccine studies conducted to date have been based on E1-deleted and thus replication-defective AdHu5 vectors [76]–[78]. Even if given at moderate doses, E1-deleted AdHu5 recombinants induce high B and CD8^+^ T cell responses in experimental animals, and in humans tested thus far. The immune responses to the transgene product of the Ad vector far surpass those achieved with other types of subunit vaccines, such as vaccinia virus recombinants or DNA vaccines [77],[78]. In a mouse model for preventative vaccination to rabies, full and long-lasting protection against a severe challenge with rabies virus could be induced with a single moderate dose of an E1-deleted AdHu5 vaccine expressing the rabies virus glycoprotein [78]. The high immunogenicity of Ad vectors relates in part to the non-cytopathic nature of such E1-deleted viruses, resulting in sustained antigen expression [78]. In addition, the vectors persist at very low levels in T lymphocytes in a transcriptionally active form [79], which allows for and supports a sustained response. Replication-competent Ad vectors, in which expression of the rabies virus glycoprotein is either driven by an endogenous Ad promoter or by the SV40 promoter inserted into the vector, which in many cell types is significantly weaker than the cytomegalovirus promoter used for replication-defective Ad vectors, have been tested in experimental animals [80],[81]. The replication-competent vectors induced protective NA titers to rabies virus in mice as well as in skunks; nevertheless, the vectors were less efficacious compared to replication-defective vectors expressing a closely related transgene product. Also, the use of replication-competent adenovirus vectors may pose risks, especially to immunocompromised individuals.

Although E1-deleted AdHu5 vectors have yielded highly promising results as vaccines in rodents, primates, and canines, the pre-existing immunity was shown to interfere with the efficacy of such vaccines [82],[83]. Nearly all adults have binding antibodies to the common serotypes of human adenovirus, and our studies showed that ∼45% of human adults in the US, ∼80% of the human population in Thailand, and up to 90% in Central Africa have detectable titers of virus NAs to AdHu5 virus [71]. We showed previously that the B cell response in pre-exposed mice could be rescued by increasing the dose of the adenoviral vaccine or by using a DNA vaccine for priming [77],[78]. Increasing the Ad vaccine dose may be problematic, as doses equal to or above 10^11^ Ad particles (vps) were shown to have systemic side effects (fever) in clinical trials [84]. Oral immunization was also shown to overcome the effect of pre-existing immunity in rodents [77], but we found that this route of immunization was not effective in nonhuman primates. It has been suggested that E1-deleted AdHu5 vectors could be used during early infancy before children become naturally infected [85]. A study conducted in India showed that only 30%–40% of infants below the age of 18 months had NAs to AdHu5 virus, and the prevalence of such antibodies rose sharply to ∼70% in children between 19 and 24 months of age. Although AdHu5 vectors were shown to be immunogenic in neonatal animals [86], their use in human infants should thus be discouraged.

In a recent phase IIb clinical trial, termed the STEP trial, AdHu5 vectors were tested as CD8^+^ T cell inducing vaccines to HIV-1 [68],[87]. The trials enrolled individuals with low or absent baseline titers of virus NAs to AdHu5 virus and individuals with moderate to high titers of such antibodies. The trials were powered to allow for testing of vaccine efficacy in reducing HIV-1 acquisition and viral set point loads. The STEP trial, which had planned to enroll 3,000 participants, was stopped and unblinded before enrollment was completed, as an interim analysis showed that the vaccine lacked efficacy and showed a trend for higher HIV-1 acquisition in vaccinated individuals that were seropositive for AdHu5 virus at the onset of vaccination. It remains to be shown whether AdHu5 vaccination of AdHu5 seropositive individuals results in an increased risk for HIV-1 acquisition. Under the assumption that the increased HIV-1 acquisition rate was related to the vaccine carrier, i.e., the AdHu5 vector rather than the HIV-1 vaccine antigens, such vectors or vectors that contain parts of AdHu5 that provide targets for NAs should not be used in humans who may be at risk for HIV-1 infections.

### E1-Deleted Ad Vectors Based on Rare Human Serotypes

Vectors based on rare serotypes of human Ads were developed to circumvent vaccine failures caused by pre-existing NAs to the vaccine carrier. Examples of such rare serotypes include AdHu35, AdHu48, AdHu11, or AdHu26 viruses, and vectors based on these serotypes have been tested for antigens of HIV-1 or tuberculosis [88],[89], but not yet for those of rabies virus.

### Ad Vectors Based on Chimpanzee-Derived Serotypes

We tested several alternative adenoviral vaccine carriers based on viruses that were isolated from chimpanzees [83], [90]–[92]. We chose isolates from chimpanzees assuming that these Ad serotypes would more closely resemble the well characterized human serotypes with regard to growth characteristics (which influences vaccine yield), tropism and receptor utilization (both of which influence immunogenicity), and transcomplementation with E1 gene products derived from AdHu5 virus. This latter point is important for logistic reasons. Mammalian cell lines that transcomplement E1 gene products of AdHu5 to E1-deleted adenoviral recombinants are available. Generation of such packaging cell lines, especially for eventual production of GMP vaccine lots, is extremely labor-intensive and time consuming. E1-deleted Ads that can be grown on the available packaging cell lines (that carry the E1 gene products of AdHu5 virus) can thus be developed much more rapidly. Using heterologous E1 gene products for transcomplementation has an added advantage; the sequences flanking the E1 gene in Ad show limited homology between different serotypes. The risk of contamination of E1-deleted adenoviral vaccine stocks with replication-competent virus that arises upon homologous recombination of the vaccine with the E1 of the packaging cell line is thus virtually absent if both are derived from different serotypes.

We tested four chimpanzee adenovirus (AdC) serotypes as vaccine carriers for the rabies virus glycoprotein. Three of those, termed AdC68, AdC7, and AdC6, can be grouped within subfamily E of human Ads and use the coxsackie-Ad receptor (CAR) for cell attachment; the other, termed AdC1, belongs to subfamily B2 and uses CD46 as its receptor for cell entry. Most humans residing in the US or Thailand do not carry NAs to the AdC viruses. Prevalence rates of NAs to AdC6 are markedly higher in some countries of sub-Saharan Africa, presumably reflecting spillover infections from chimpanzees [71]. AdC vectors were found to be highly immunogenic in mice and nonhuman primates. As was shown for human serotype Ad vectors, the CAR-binding AdC vectors outperformed the CD46-binding AdC1 vector.

The immunogenicity of the E1-deleted AdC vaccine carriers is not strongly impaired by pre-existing immunity to common human serotypes of Ad, at least in mice [92]. When tested as vaccine carriers for the rabies virus glycoprotein, the AdC vectors were shown to induce sustained and protective titers of rabies virus NAs after a single dose in mice. A single dose of the vaccines induced protective titers of NAs after intramuscular, oral, or intranasal immunization in adult as well as neonatal mice [83],[93],[94]. Titers were sustained in mice and showed no decline within the first 20 months after immunization (Z. Q. Xiang and H. C. Ertl, unpublished data). Additional studies are needed to confirm the longevity of protective responses in other species.

### Ad Vectors Based on Dog-Derived Serotypes

Replication-defective vectors based on canine Ad serotype 2 have been generated. Upon oral immunization with high doses of the vector given in baits, most of the dogs seroconverted [95]. Although titers induced by the vaccines were low, they were remarkably sustained, yielding protection to all of the seroconverted dogs for at least 2 years. It should be pointed out that canine Ad2 is one of the causative agents of kennel cough and a component of available vaccines used for vaccination of puppies. The use of such vectors may not be optimal for immunization of dogs that may be pre-immune to canine Ad2 due to vaccination or natural infections.

A replication-competent Ad vector from the canine Ad serotype 2 was tested in pigs in comparison to one dose of a commercial vaccine [96]. Pigs immunized orally or intranasally failed to develop antibodies to rabies virus, even at a high dose of the virus. Upon systemic immunization with the Ad vector, titers came up markedly slower and were lower than upon immunization with the commercial rabies vaccine. It should be pointed out that a replication-competent canine Ad vector might not replicate in swine, even though porcine and canine Ads are closely related. Lack of replication in swine, which may explain the disappointing immunogenicity of the replication competent vector, was not formally tested for, but was suggested by the complete lack of shedding of virus upon oral or intranasal application.

### Ad Vectors Based on an Avian-Derived Serotype of Adenovirus

One group tested an Ad vector derived from an avian serotype, i.e., Celo virus, as a vaccine carrier to elicit an anti-rabies immune response in mice [97]. Repeated vaccination protected less than 50% of the mice, indicating that such vectors lack the high immunogenicity of Ad vectors isolated from mammals.

## Summary

Clearly, the global pipeline for new rabies vaccines looks impressive at first glance, although it should be pointed out that most of these novel vaccines have not yet undergone extensive pre-clinical testing or comparison to commercially available vaccines using standardized reference sera to measure neutralization titers, let alone clinical testing. Rabies is a nearly always fatal disease that still kills more than an estimated 50,000 people each year and inflicts costs in the millions of dollars, but in spite of this remains an orphan disease. Rabies is a disease that affects mainly the poorest of the poor. Developed countries with resources for research and development of novel vaccines do not view rabies as a priority, for after all an efficacious vaccine is available to those that can afford it. The vaccine industry in developed countries has little incentive to develop a new vaccine to rabies for resource-poor countries, and the fledging vaccine companies in developing countries lack the infrastructure to generate novel vaccines, and are thus focused on the manufacture of traditional vaccines.

Notwithstanding this, human rabies vaccines are needed for two applications: vaccines for large-scale preventative vaccination of children in resource-poor countries with a high incidence rate of rabies, and vaccines for PEP for use after exposure to a rabid animal. Currently, the same vaccines are used for both applications, but one could well envision the use of different vaccines and vaccine prototypes to address these critical objectives. A vaccine for preventative vaccination given along with other vaccines to children between the ages of 1 and 2 years would need to induce sustained protection for at least 10–20 years after a single dose. Such a vaccine would have to be inexpensive, safe, and acceptable to parents. The only vaccines that could potentially induce such a sustained response after a single immunization are those based on Ad vectors. To avoid interference with endogenous NAs to the vaccine carrier, Ad vectors should be based on rare human serotypes or on nonhuman serotypes such as those from chimpanzees.

For PEP, vaccines need to rapidly induce protective titers of rabies-specific NAs. An improved PEP rabies vaccine should be inexpensive and reduce the number of doses from the currently used four to five dose regimens. Ideally, such a vaccine would also require less or no RIG. Vaccines based on gene transfer technology inevitably delay immune responses, as they require initial transcription and translation before the vaccine antigen is expressed. DNA vaccines and viral recombinant vaccines may thus not be optimal for PEP. Better adjuvants would increase the immunogenicity of current vaccines and should clearly be pursued in more depth with the new vaccines in development. Attenuated rabies mutants, in which crucial genes are deleted and replaced with a second glycoprotein gene, were shown pre-clinically to induce more potent immune responses than traditional vaccines and responses came up more rapidly. Such viral mutants may be highly suited to replace the current vaccines for PEP.

**Key Learning Points**

- High incidence rates of rabies in developing countries
- Reasons for the ongoing high human fatality rates for rabies
- Correlates of protection against rabies virus
- Post-exposure versus preventative vaccination to rabies virus
- Different vaccine prototypes for rabies in pre-clinical development

**Key Papers**

1. Warrell MJ (2003) The challenge to provide affordable rabies post-exposure prophylaxis. Vaccine 21: 706–709.
2. Ichhpujani RL, Mala C, Veena M, Singh J, Bhardwaj M, et al.(2008) Epidemiology of animal bites and rabies cases in India. A multicentric study. J Commun Dis 40: 27–36.
3. Cenna J, Tan GS, Papaneri AB, Dietzschold B, Schnell MJ, et al. (2008) Immune modulating effect by a phosphoprotein-deleted rabies virus vaccine vector expressing two copies of the rabies virus glycoprotein gene. Vaccine 26: 6405–6414.
4. Wang X, Bao M, Wan M, Wei H, Wang L, et al. (2008) A CpG oligodeoxynucleotide acts as a potent adjuvant for inactivated rabies virus vaccine. Vaccine 26: 1893–1901.
5. Xiang Z, Gao G, Reyes-Sandoval A, Cohen CJ, Li Y, et al. (2002) Novel, chimpanzee serotype 68-based adenoviral vaccine carrier for induction of antibodies to a transgene product. J Virol 76: 2667–2675.
